# Clinical Trial Authorisation: A Final Look Back to Better Appraise the New European Regulation

**DOI:** 10.2174/1574887118666230320124012

**Published:** 2023-08-15

**Authors:** Stéphane Vignot, Gaëlle Guyader, Valérie Salomon, Philippe Vella, Isabelle Yoldjian, Patrick Maison, Christelle Ratignier-Carbonneil

**Affiliations:** 1 Agence Nationale de Sécurité du Médicament et des Produits de Santé, Boulevard Anatole France, 93200, Saint Denis, Grand Paris, France

**Keywords:** Clinical trials, regulatory science, access to innovation, European regulation, authorisation, indicators

## Abstract

The implementation of the new European Clinical Trial Regulation on 31 January 2022, is a major step to promote clinical research in Europe. The French National Agency for Medicines and Health Products Safety (ANSM) proposes to share some key aspects of the preparation for the application of the Regulation initiated in 2017 and to discuss shared indicators that should be considered to monitor clinical trials opportunities on a territory with regards to access to innovation for patients and attractiveness for sponsors. New criteria based on the time from the first request for authorisation to the first inclusion could be of particular interest to appraise the implementation of the European Clinical Trial Regulation.

## 1 INTRODUCTION

The European Regulation on clinical trials on medicinal products for human use (EU CTR) was implemented on 31 January 2022, opening a new era for the conduct of clinical trials in Europe. Authorisation decisions remain national, but the new regulation allows for joint assessments between the concerned Member States within a harmonised timeframe [1]. National procedures for trials on medicinal products may continue for a period of one year, with the sponsors having the choice of submitting their application under the framework of the outgoing Directive 2001/20/EC or under the scope of the new Regulation. At the end of this transitional period, all new applications will be assessed under the new Regulation. The Regulation aims to ensure that Europe offers a more attractive environment for clinical research. Stakeholders have prepared for the implementation of the regulation in this spirit. The notion of attractiveness has been of particular concern to the ANSM, the national competent authority (NCA) in France. The number of trials is an indicator often used by stakeholders to assess the attractiveness of clinical research, and therefore the possibilities for patients to access innovation [2]. Indeed, the number of authorisations of clinical trials on medicinal products decreased by 23% between 2015 and 2017 in France (from 928 to 713 applications). This evolution concerned especially early trials and could be explained by an understandable level of uncertainty around clinical trials following the death of a healthy volunteer during a phase I study in 2016 [3]. The ANSM has therefore set up an action plan to optimise its clinical trial assessment procedures in order to remove potential obstacles before the European regulation is implemented.

We propose to outline the main components of the action plan, present a critical analysis of its impact on the criterion of the number of trials being submitted and then discuss the common indicators that should be considered to monitor territorial opportunities for the conduct of clinical trials following the implementation of the European Clinical Trial Regulation.

## 2 ANSM ACTION PLAN ON CLINICAL TRIAL ASSESSMENT

During the initial assessment of a clinical trial, the competent authority must verify that the safety of the participants will be ensured and that they will not be exposed to an unreasonable risk or loss of opportunity compared to the standard of care. These principles remain the basis for any optimisation of the clinical trial authorisation process.

The measures implemented by the ANSM are:

• Harmonisation of assessment doctrines according to the clinical context of the request, and to the previous knowledge of the investigational medicinal product, where appropriate. The objective was not to define all new guidelines but to define internal processes that ensure that guidelines are well-known, shared and correctly applied. For example, patients with a life-threatening condition may have a higher benefit and a lower risk with respect to trying new drugs [4]. Some guidelines take into account this risk-mitigated approach, especially for non-clinical and quality parts of the dossier such as ICH S9 guideline which proposes clear recommendations in the assessment of clinical trials for the treatment of patients with advanced disease and limited therapeutic options [5]. It also recommends that the NCA adapts these guidelines for less advanced stages. Internal harmonisation is then crucial to guarantee a standardized assessment. Such harmonization is also important for assessors to avoid unnecessary, and potentially repetitive, internal discussions and to ensure that they can rely on a consistent and secure approach in light of various clinical contexts.

• Strengthening of collegiality. Exchanges between assessors are crucial for shared risk-benefit analysis, identifying and challenging grounds for non-acceptance, sharing harmonized practices, and continuously updating internal knowledge. In this regard, multidisciplinary meetings in cancer care have been a particularly inspiring example as an experience of inter-speciality communication in order to be consistent with the best available evidence [6]. The need for collegiality can be identified either as soon the application is received (for example for early phase trials or trials involving vulnerable population) or during the assessment, if assessors identify a specific issue. Internal collegiality is favoured over requesting external expertise in order to avoid delaying the assessment of the application. External experts are preferably consulted for the reassessment of internal guidelines.

• Simplification measures. We standardised the wording of the requests for further information (RFI) and of the grounds of non-acceptance in order to clarify and simplify the exchanges with the sponsors. These exchanges are now conducted in English in order to avoid translation difficulties that might occur during the discussion with sponsors (many misunderstandings resulted from mistakes in the successive French to English translation of the questions and English to French translation of the sponsor responses).

Two additional initiatives have to be mentioned:

• The ANSM created an early trials unit was created in late 2017 in order to solidify internal expertise with specialized assessors (non-clinical, quality, clinical and safety), medical-scientific advisors, and systematic collegiality within the unit [4].

• Since 2018, the sponsors can opt for Fast Track procedures in order to accelerate patients’ access to innovative medicines in clinical trials. This procedure is dedicated to patients with acute medical needs in fields such as paediatrics, oncology-haematology, rare diseases, and early phases or to confirmatory trials for a molecule or combination that has already been evaluated in the same context (the request for information or decision is sent between day 14 and 21 after submission) [7]. These pre-existing procedures proved useful during the COVID pandemic for trials related to the management of SARS-Cov2 infection or its complications.

At the same time, the coordination with French Ethics Committees has been improved thanks to a Pilot Project promoting systematic dialogue with ANSM and to the specialisation of some of the French Committees in the assessment of European trials on the medicinal product. Similarly, exchanges with Member States Competent Authorities have been strengthened in order to prepare jointly for the implementation of the European Clinical Trial Regulation, in particular through European groups (Clinical Trial Facilitation Group becoming Clinical Trial Coordination Group in 2022; Clinical Trial Expert Group and Clinical Trial Action Group under the responsibility of the European Commission).

## 3 ANSM METRIX 2017 - 2021

The number of clinical trial applications for medicinal products submitted annually to ANSM was collected in an internal database. All applications were considered regardless of the final decision (authorisation, refusal, or withdrawal by the sponsor). Year 2017 was chosen as a reference since it is the year when the need for optimising clinical trial assessment was established in France and as it allows to discuss a before/after evaluation of the above action plan. Trials concerning Advanced Therapy Medicinal Product (ATMP) have been isolated as different timelines and assessment processes apply. Early-phase trials have been isolated as they were considered particularly sensitive for clinical research activity in France.

Data on ANSM authorisation of medicinal product clinical trials are presented in Table **1** and Fig. (**1**). Between 2017 and 2021, the number of submissions increased by +23%. An increase in sponsor withdrawals was observed in particular in 2020 due to the COVID-19 pandemic. The impact of this health crisis led some sponsors to reconsider their projects while some trials related to the management of COVID were abandoned during the assessment due to the rapid evolution of knowledge on COVID-19, in particular between March and June 2020 [8]. Despite this situation, a regular increase in the total number of authorised trials is observed, by 15% between 2017 and 2021 (741 to 855 trials). This evolution is notably visible for early trials (+10%).

## 4 DISCUSSION: INDICATORS TO BE CONSIDERED UNDER CLINICAL TRIAL REGULATION

The increase in the number of authorised trials could lead to believe that the ANSM action plan has achieved its main objective by halting the decline in the number of new authorised trials in France. Do these results reflect an improvement in attractiveness and access to innovation?

Indicators that could be discussed are presented in Table **2**. The raw number of clinical trials aggregates very different typologies of trials, thus imperfectly translating the notion of access to innovation. The situation of early trials is particularly emblematic here. The organisation of health establishments in France has led to a high degree of specialisation of centres in early trials including sick volunteers, particularly in onco-haematology. The conduct of this kind of trial is facilitated, leading to a strong representation of this category of trials in the requests for authorisation, compared to phase I trials in healthy volunteers, which are in minority. In practice, 70 to 80% of early trials conducted in France concern onco-haematology. A particularity of these trials is that they regularly include expansion cohorts in simple (a single molecule or a single combination evaluated in one indication) or complex (multiple combinations, multiple indications) designs [9]. Concerns for access to innovation are undoubtedly different for a patient who is likely to participate in such an early trial compared to the situation of a healthy volunteer who might participate in an early trial that is part of the broader development of a drug. Defining the attractiveness of a country based on the absolute number of trials might lead to overlooking the fact that trials of different types, from phase I to phase IV, have vastly different numbers of participants, ranging from a few dozen to several thousands and different designs.

During the same period, the number of substantial modifications of clinical trials increased by 38%. The number of substantial modifications reflects a level of overall clinical research activity if we consider that a trial with a request for a substantial modification is an active trial, but the absolute number combines modifications of very different impacts, ranging from an update of documents to the addition of a new treatment cohort in a complex design trial.

The absolute number of trials and substantial modifications are therefore not reliable indicators for monitoring the opportunities for access to innovation through clinical research and the attractiveness of a country or geographical area.

Concerning the follow-up of assessment delay, the first expectation for authority is compliance with its regulatory obligations. It can also be considered in France as a marker of the sensitivity of the clinical trial process to organisational constraints. The implementation of the action plan was immediately accompanied by a decrease in the average decision time of the ANSM (from 62 to 45 days) but the COVID-19 crisis has led to a 2-week increase in this average time in 2021. Continuous efforts are needed to ensure that timelines are respected for the implementation of the European Clinical Trial Regulation. It is important to remember that the objective of the Regulation is not to reduce assessment delays but to promote the harmonisation of the clinical trial assessment process in Europe and the predictability of application processing timelines. From a sponsor standpoint, the overall reliability of the clinical trial authorisation process could be a strong argument for the attractiveness of the European Union.

The dynamics of clinical research could be reflected by the number of patients to be included in trials. From a patient’s perspective, a high number of available slots for inclusion in clinical trials can be considered an indicator of opportunities for patients to access innovation when considered at a national level (or for a specific geographic area). This macroscopic vision, which appears more accurate at first glance than the number of trials, has certain limitations. The definition of access to innovation remains imprecise [10, 11] and relying on this indicator reflects the assumption that any enrolment in a clinical drug trial is part of an innovation process, which is not always accurate. Limiting the indicator to trials involving molecules that do not have marketing authorisation would be an overcorrection as some trials that evaluate new indications or new combinations of molecules that have a marketing authorisation can also be considered as opportunities to access innovative strategies. Monitoring a balanced indicator is technically more difficult for international comparisons or retrospective analyses. In addition, the number of included patients and the ratio of included patients to the total number of patients to be included are interesting post-trial indicators for assessing the capacity of a health system to commit to clinical research. They may represent an indicator of attractiveness for sponsors in their choice of areas in which to deploy their clinical trials, but they do not represent the overall capacity for the patient to access innovation.

The time between submission of the clinical trial application and inclusion of the first patient could represent an interesting indicator for evaluating the capacity of a health system to implement clinical research efficiently and therefore to offer the best possibilities of access to innovation for patients and to present the best attractiveness for sponsors. It is a composite indicator that integrates the performance of the authorities in the assessment of the initial application, the quality of the sponsor's interactions with the authorities and then with the investigating centres for the implementation of the trial, and finally, the investigators' capacity to identify and include patients.

Finally, participating in a clinical trial can be a way to access innovation as discussed above and being part of a research dynamic is important for patients, caregivers and institutions. However, the completion of a clinical trial should not be seen as an end in itself. It is also important to consider the impact that the results may have on practice. This impact could initially be estimated according to the relevance of the research question and the underlying public health issue (large target population, unmet medical need, evaluation of a potentially breakthrough innovation versus confirmatory study). The impact of a study's results also needs to be judged by the timing of the release of the data compared to the evolution of practices: a study with a high potential impact at the time of its initial authorisation may become less relevant once the results have been analysed if the strategies and scientific context have changed in the meantime. The quality of the collected data will also be an important element in determining the potential impact of a study. Including large numbers of patients in clinical trials without guaranteeing relevant and monitored data collection could lead to reserves in the analysis of the results and therefore to their subsequent use to make changes to clinical practice. These elements deserve to be integrated into the initial considerations of the conception of a clinical trial. Discussions on decentralised trials have been conducted in Europe to encourage the use of remote procedures for information, inclusion, follow-up of patients and data monitoring. The concerted proposals between the European National Competent Authorities, the European Commission and the EMA are based on the lessons learned from the initial period of the COVID pandemic [12]. The objective of the recommendations is to facilitate the conduct of clinical trials and to accelerate enrolment. It will be up to the NCAs to check that the decentralised conditions of patient follow-up are not to the detriment of the safety of the participants. The sponsor should also take care to anticipate the questions that could be raised when interpreting the results of the study (compliance, reliability of toxicity data, *etc*.). Conducting an efficient clinical trial is not limited to quickly reaching the inclusion objective but also to obtaining useful data for analysing our practices. Beyond the number of trials or the number of inclusions, indicators of the quality of trials deserve to be discussed, in the design of trials and the interpretation of their results.

## CONCLUSION

Promoting access to innovation through efficient clinical research activity relies on a multi-step process. The reliability of authorisation processes is necessary and the implementation of the clinical trials regulation provides a unified response to this challenge. It remains essential to continue to work on improving the quality of submitted applications (how to be ready for submission) as well as on the opening of centres and the selection of patients (how to anticipate inclusions). The indicator of “time from First request for authorisation to First inclusion” could be considered as the result of a collective momentum and this is what we, authorities, sponsors, investigators, patients and general public, can expect for the progress of clinical research in Europe.

## Figures and Tables

**Fig. (1):**
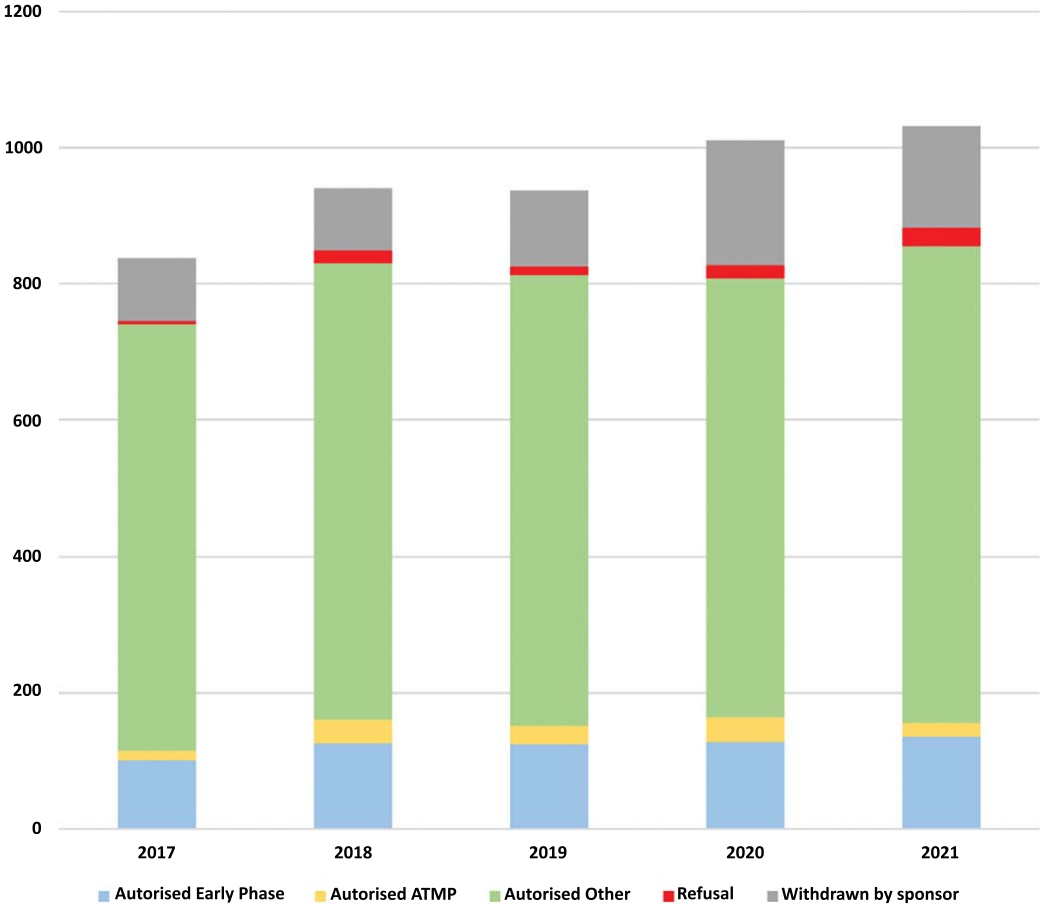
Clinical trials on medicinal products submitted to ANSM between 2017 and 2021. **Abbreviation:** ATMP: Advanced therapy medicinal products.

**Table 1:** Submission and authorisation of clinical trials on medicinal products by the ANSM between 2017 and 2021: initial applications and substantial modifications.

**-**		**2017**		**2018**		**2019**		**2020**		**2021**	
**-**		**N**	**%**	**N**	**%**	**N**	**%**	**N**	**%**	**N**	**%**
Initial request	Submission	838	-	940	-	938	-	1011	-	1032	-
	Autorisation	741	88%	830	88%	813	87%	809	80%	855	83%
	Refusal	4	0, 5%	19	2%	12	1%	18	2%	28	3%
	Withdrawnal	93	11%	91	10%	113	12%	184	18%	149	14%
Subsantial Modif.	Submission	2682	-	3022	-	3863	-	4085	-	3712	-
	Autorisation	2632	98%	2885	95%	3700	96%	4017	98%	3618	97%
	Refusal	2	0, 1%	6	0, 2%	13	0, 3%	13	0,3%	8	0, 2%
	Withdrawnal	48	2%	131	4%	150	4%	55	1%	86	2%

**Table 2:** Potential indicators of access to innovation for patients in clinical trials and of attractiveness for sponsors.

**Indicators**	**Comments**
Number of clinical trial authorisations	Heterogeneous view of access to innovation and attractiveness as applications are heterogeneous either in size and scope.
Number of substantial modifications	Reflecting the activity of the trials (*i.e*. “living trials”) but heterogeneous view of access to innovation and attractiveness as modifications are heterogeneous
Assessment delay	Predictability and reproducibility of delays: a factor for attractiveness of the European Union
Number of patients to be included	Opportunity for patients to be treated: indicator of access to innovation
Number of included patients or Ratio of included patients to patients to be included	Reflect the ability to successfully conduct a clinical trial: indicator of attractiveness for sponsors in a geographic area
Delay from request for authorisation to inclusion of the first patient	Reflects the ability to successfully implement a clinical trial: indicator of access to innovation for patients and of attractiveness for sponsors

## LIST OF ABBREVIATIONS

NCA: National Competent Authority
ATMP: Advanced Therapy Medicinal Product
